# Interplay between Hormones, the Immune System, and Metabolic Disorders

**DOI:** 10.1155/2018/8654212

**Published:** 2018-09-30

**Authors:** Francisco J. Rios, Naïma Moustaïd-Moussa, Joilson O. Martins

**Affiliations:** ^1^Institute of Cardiovascular and Medical Sciences, BHF Glasgow Cardiovascular Research Centre, University of Glasgow, UK; ^2^Department of Nutritional Sciences and Obesity Research Cluster at Texas Tech University, Lubbock, USA; ^3^Department of Clinical and Toxicological Analyses, School of Pharmaceutical Sciences, University of São Paulo, São Paulo, Brazil

This special issue of Mediators of Inflammation focuses on diverse research areas related to the interplay between hormones, immune response, and metabolic disorders.

Hormones are metabolic components produced by different cell types, capable of regulating body homeostasis and the cross talk among the endocrine, cardiovascular, and immune systems. In patients with compromised immune response, inflammation may last longer or may be ineffective, leading to recurrent infections or other types of systemic dysfunctions associated with chronic inflammation. In the past few years, it became evident that hormones, neurotransmitters, and dietary factors are specific modulators of cells from the immune system by fine-tuning their activation and key functions. Of note, cells from the immune system present high expression of receptors for different hormones present in the blood circulation, such as aldosterone and glucocorticoids. This in turn might also affect the vascular function leading to cardiovascular diseases. Therefore, the main scope of this edition is to contribute to knowledge in this growing and innovative area, through reviews and original articles that will help to understand the diverse mechanisms by which hormones and/or diet can influence inflammatory response and immune activation.

This special issue covers the most current research aimed at elucidating how metabolites and dietary components such as vitamins, bioactive compounds, or lipid mediators influence inflammatory processes. Articles published in here explore the cell and molecular mechanisms underpinning the endocrine/paracrine networks of regulatory immune mediators, their targets on immune cell signaling, and how they contribute to metabolic dysregulations in obesity. The current issue also highlights the importance whereby hormones contribute to cellular homeostasis and immune system regulation, and that an imbalance in this well-regulated system can lead to cardiovascular and/or metabolic disorders.

In this edition of Mediators of Inflammation, N. V. Fedorova et al. studied the effects of insulin, glucagon, and 17*β*-estradiol (E2) on the activation of human neutrophils. They reported that hormones influence the activation of neutrophils and induce their adherence to blood vessels in diabetes and metabolic disorders. Extracellular matrix proteins play a crucial role in this process. Moreover, glucagon can contribute to the development of metabolic vascular disorders by initiating the secretion of cathepsin G, an important enzyme present in neutrophils with bactericide activity. In addition, cathepsin G may promote inflammatory response and stimulate further neutrophil adhesion via proteolysis of cell surface receptors. On the other hand, insulin and E2 can alter the adhesion of neutrophils initiating the secretion of metalloproteinases, which modify extracellular matrix proteins.

In newborns, the developing hypothalamic-pituitary-adrenal axis is activated after exposure to painful and stressful situations as a result of increased glucocorticoid secretion by the adrenal gland cortex. A high concentration of cortisol might also result in an increased risk factor for insulin resistance, hyperlipidaemia, immunologic deficiencies, and destructive changes in the hippocampus. G. De Bernardo et al. presented a pilot but interesting study showing that a full-time rooming-in (for 24 hrs.) is better than a partial rooming-in (for 14 hrs.) in reducing neonatal stress response in hospitalized newborns. This was supported by their data showing that lower salivary cortisol levels (SCLs) may have long-term positive effects in reducing the risk of metabolic syndrome, high blood pressure, and cognitive and behavioural changes. In addition, in an experimental study, A. M. Balbino et al. evaluated long leptin receptor isoform (ObRb) expression in lung endothelial cells from low birth weight (LBW) rats and examined the production of lipid mediators and cytokines. They found that lung endothelial cells isolated from intrauterine undernourished rats with a LBW exhibit suppressed IL-1*β* and IL-6 production after applying inflammatory stimuli. They further demonstrate that these effects may be linked to a lack of ObRb receptor expression and mediated in part by the NF-*κ*B and p38 MAPK signaling pathways.

Wound healing involves a series of tightly controlled biochemical and cellular events, divided in 3 concomitant and overlapping phases: inflammation, proliferation, and remodeling. Poor wound healing or chronic wounds are characterized by a full thickness in depth and a slow healing tendency. Examples of these include diabetic foot ulcers, venous leg ulcers, and pressure ulcers, and all represent a silent epidemic that affects a large fraction of the world population and represent a major public health problem. J. R. Silva et al. reported that omega-6 (*ω*-6) fatty acids can improve the wound healing process by modulating cellular responses, through increased endothelial and inflammatory cell migration and function as well as enhanced angiogenesis at the wound site, therefore accelerating the wound healing process.

Sex hormone-binding globulin (SHBG) is a serum protein released mainly by the liver, and a low serum level correlates with a higher risk for metabolic syndrome including diabetes, obesity, and cardiovascular events. H. Yamazaki et al. report that SHBG exhibits anti-inflammatory effects involving macrophages and adipocytes, as evidenced by suppressed mRNA levels for inflammatory cytokines such as IL-6, TNF*α*, and MCP-1, all known to be highly expressed in adipocytes, with major effects on the chronic low grade inflammation. Additionally, in a review article M. Mendes-Braz and J. O. Martins discuss recent publications addressing the effects of diabetes mellitus (DM) on oxidative stress response and inflammatory processes, which play an essential role in ischaemia-reperfusion injury and impaired hepatic regeneration after liver surgery. Authors highlight the need to expand the knowledge in this area, to benefit patients with DM who undergo surgical procedures that are increasing in clinical practice.

T. Zhu et al. showed that in lipopolysaccharide- (LPS-) induced pulmonary inflammation, there is a reduction in the surfactant protein-A (SP-A) expression, both *in vivo* and *in vitro*. Pulmonary surfactant (PS) is synthesized by type II alveolar epithelial (ATII) cells and plays a crucial role in the maintenance of pulmonary compliance and fluid balance in the lungs, in preventing the lung from collapsing at the end of expiration, and in regulating the size of alveoli and pulmonary immune defenses. They also reported that the SP-A-enhancing property of liraglutide was most likely mediated via the thyroid transcription factor-1 (TTF-1) signaling pathway.

*Leishmania* is an obligatory intracellular protozoan that is transmitted vectorially and causes a disease that affects two million people globally each year. This infection results in lesions on the skin, mucosa, or viscera, depending on the specie of the parasite as well as the host response. L. C. Reis et al. reported new insights into the immunology of leishmaniasis by showing that IGF-I is an effector element in cellular responses mediated by IL-4, leading to M2 macrophage polarization. Mechanisms underlying these effects are dependent on the PI3K/Akt pathway during *L. major* infection. In addition, they speculated that individuals in endemic areas might be more susceptible or resistant depending on the expression of basal IGF-I.

Histamine is a biogenic vasoactive amine, which may impact the immune system activation, by acting as a regulatory component to establish homeostasis after injury or preventing the inflammatory process. Histamine is the main mediator responsible for the clinical symptoms in type 1 hypersensitivity reactions and has pleiotropic effects that are dependent on the interaction with histamine receptors. A. C. C. C. Branco et al. discuss recent findings about histamine effects on inflammation through the activation of intracellular pathways to enhance the production of inflammatory mediators and cytokines in different immune cells. In addition, a review article written by L. M. Oliveira et al. discusses the effects of vitamin A on the innate and adaptive immunity with special emphasis on the inflammatory status, which is becoming a public health concern. Currently, more than 2 billion people are affected by micronutrient deficiency worldwide and vitamin A supplementation is highly effective in reducing mortality from different causes, such as intestinal diseases, neurodegenerative processes, skin aging, and cancer.

In summary, we hope that the original research and the review articles featured in this special issue will enhance the knowledge about the importance of different endocrine systems in inflammatory processes, and help shed light on potential avenues for the development of novel therapies for increasingly prevalent inflammatory and metabolic diseases.

